# Awareness of Tuberculosis Among General Population of Riyadh, Kingdom of Saudi Arabia: A Cross-Sectional Study

**DOI:** 10.7759/cureus.80900

**Published:** 2025-03-20

**Authors:** Maram S Alshammari, Talal M Hafiz, Eyesha Junaidallah, Hessa M Alanazi, Haya Aldawood, Danh S Aldhupiapan, Sana A Sankari, Maha M Faraidy, Amirah Alnaser, Wasif Ali Khan

**Affiliations:** 1 College of Medicine, Almaarefa University, Riyadh, SAU; 2 Medicine, King Abdulaziz Medical City, Riyadh, SAU; 3 Basic Medical Sciences, Almaarefa University, Riyadh, SAU

**Keywords:** awareness, riyadh, saudi arabia, tb, tuberculosis

## Abstract

Background: Tuberculosis (TB) continues to pose a significant global health burden despite advances in treatment and prevention. Awareness and knowledge of TB among the general population are critical for effective disease control, especially in regions with a moderate incidence like Riyadh, Saudi Arabia.

Objective: This study aims to assess the knowledge, attitude, and practice toward tuberculosis among the general population of Riyadh, Kingdom of Saudi Arabia (KSA).

Methods: A cross-sectional survey was conducted from May to June 2023, targeting 392 adult residents of Riyadh. A structured questionnaire assessed demographic details, knowledge of tuberculosis transmission, symptoms, diagnosis, prevention, and treatment, as well as attitude, and practice toward the disease. Descriptive and bivariate analyses were performed using SPSS.

Results: Among the 392 participants, 94.6% had heard of tuberculosis, with 85.2% aware that tuberculosis affects the lungs. However, knowledge about tuberculosis transmission and its bacterial cause was lacking in 14.5% and 31.1% of respondents, respectively. Significant associations were found between tuberculosis awareness and marital status (p=0.001) as well as occupation (p=0.001). Health providers had the highest awareness, while younger participants and housewives demonstrated lower awareness levels.

## Introduction

Tuberculosis (TB) remains a significant public health issue worldwide, ranking among the top 10 causes of death despite considerable advances in medical research and public health initiatives [1]. The World Health Organization reports an estimated 10 million new tuberculosis cases and approximately 1.4 million deaths annually, indicating the persistent threat posed by this infectious disease [1,2]. In Saudi Arabia, tuberculosis continues to be a public health concern with an estimated incidence rate of 14 per 100,000 population [3]. Riyadh, as the capital city, is no exception to this burden. Understanding the level of awareness and knowledge about tuberculosis among the general population is crucial for effective prevention and control measures [4,5].

Numerous studies have been conducted globally to assess tuberculosis awareness, revealing varying levels of knowledge and misconceptions about the disease in different communities [6,7]. In Saudi Arabia, several studies have evaluated tuberculosis awareness among specific populations, such as healthcare workers, students, and patients [8,9]. However, comprehensive data on tuberculosis awareness among the general population in Riyadh are limited, underscoring the need for research in this area. Similar studies conducted in other regions, including Oman and Southeast Asia, have highlighted the importance of public awareness in the fight against tuberculosis [10,11]. A study in Oman demonstrated that increased knowledge about tuberculosis could lead to better health-seeking behaviors and adherence to treatment protocols [10]. Another study in Southeast China identified gaps in public knowledge about tuberculosis, emphasizing the need for targeted educational interventions to enhance disease awareness [12].

Furthermore, research from Pakistan and Lesotho suggests that awareness campaigns can significantly impact the general population's perception and understanding of tuberculosis, leading to more proactive health-seeking behavior [13,14]. Therefore, this study aims to assess the level of tuberculosis awareness, perceptions, and attitudes among the general population of Riyadh in 2024. By identifying knowledge gaps and barriers to accessing tuberculosis care, the findings can inform future public health interventions and policies to improve tuberculosis prevention and control efforts in Riyadh and beyond. This research seeks to contribute valuable insights into the current state of tuberculosis awareness, ultimately aiding policymakers in formulating effective strategies to combat the disease in Saudi Arabia.

## Materials and methods

Study design and setting

This study is a cross-sectional survey conducted in Riyadh, the capital city of the Kingdom of Saudi Arabia. The target population comprises adult males and females aged 18 years and above residing in Riyadh during the study period.

Sampling method

A quota sampling technique was employed to recruit a representative sample of 392 adults, inclusive of both genders and all nationalities living in Riyadh. Individuals below the age of 18 years or not residing in Riyadh were excluded from the study.

Data collection tool

A structured questionnaire was developed based on existing literature and expert consultation to assess the participants' knowledge, attitudes, and practices toward tuberculosis. The questionnaire included four main sections. The first section collected demographic information such as age, gender, nationality, educational level, and occupation. The second section evaluated participants’ awareness about tuberculosis symptoms, transmission, risk factors, and treatment options (Appendix A). The third section focused on perceptions and attitudes toward tuberculosis patients and the disease itself. Finally, the fourth section identified potential challenges faced in accessing tuberculosis care and treatment.

Data collection process

Seven trained data collectors conducted face-to-face interviews with participants in various public places, including malls, restaurants, cafes, and parks, from May to June 2024. The questionnaire was administered in both Arabic and English to accommodate all participants.

Data analysis

The collected data were coded, cleaned, and entered into SPSS software (version 25) for statistical analysis. Descriptive statistics, including frequencies and proportions, were calculated for all variables. Bivariate analysis was performed to identify associations between demographic factors and levels of TB awareness. A scoring system was developed to assess the level of awareness, with scores categorized into low, moderate, and high awareness levels based on participants’ responses.

Ethical considerations

Ethical approval for this study was obtained from the Institutional Review Board (IRB) at Almaarefa University (IRB No: IRB23-053). All participants provided informed consent before participation, and data confidentiality was strictly maintained.

## Results

The demographic distribution of the participants shows that the majority were female, 223 (56.9%), while males constituted 169 (43.1%) participants. The majority of respondents were Saudi nationals, 245 (62.5%), with non-Saudis comprising 147 (37.5%) participants. The majority of participants were aged 40 years and older (334, 66.5%), followed by those aged 30-39 years (107, 21.3%), 23-29 years (13, 2.6%), and 18-22 years (4, 0.8%). Regarding marital status, 171 (43.6%) of participants were single, 168 (42.9%) were married, 28 (7.1%) were divorced, and 25 (6.4%) were widowed. In terms of occupation, 96 (24.5%) were health providers, followed by 108 (27.6%) categorized as "other." Housewives accounted for 80 (20.4%), engineers 57 (14.5%), and business people 51 (13%) as shown in Table 1.

**Table 1:** Shows demographic characteristics of the participants. Demographic characteristics of the study participants, including gender, nationality, age, marital status, and occupation. The data is presented as frequency (n) and percentage (%).

Parameters		Frequency	Percentage
Gender	Male	169	43.1
	Female	223	56.9
Nationality	Saudi	245	62.5
	Non-Saudi	147	37.5
Age	18-22	4	0.8
	23-29	13	2.6
	30-39	107	21.3
	40+	334	66.5
Marital status	Single	171	43.6
	Married	168	42.9
	Divorced	28	7.1
	Widow	25	6.4
Occupation	Housewife	80	20.4
	Engineer	57	14.5
	Health provider	96	24.5
	Business	51	13
	Other	108	27.6

The majority of participants (371, 94.6%) reported being aware of tuberculosis, while 21 (5.4%) indicated that they were not aware. Regarding whether tuberculosis is an infectious disease that usually affects the lungs, 334 (85.2%) were aware, while 58 (14.8%) were not. When asked if *Mycobacterium tuberculosis* bacteria is the cause of tuberculosis, 270 (68.9%) knew the correct answer, whereas 122 (31.1%) were not aware. A majority of participants, 347 (88.5%), recognized that a cough is a symptom of tuberculosis, while 45 (11.5%) did not. Additionally, 335 (85.5%) were aware that tuberculosis can spread from one person to another, while 57 (14.5%) were unaware. Regarding diagnosis, 314 (80.1%) knew there was a special test for tuberculosis, but 78 (19.9%) were not aware. When asked if tuberculosis is curable, 304 (77.6%) affirmed, while 88 (22.4%) did not know. Knowledge of the Bacillus Calmette-Guérin (BCG) vaccine preventing tuberculosis was present in 296 (75.5%) participants, with 96 (24.5%) unaware. Awareness that the lungs are the most commonly affected organ was reported by 339 (86.5%) participants, while 53 (13.5%) were unaware. A total of 275 (70.2%) were aware that tuberculosis can also affect other organs, such as the lymph glands and bones, while 117 (29.8%) were not. Moreover, 320 (81.6%) participants knew tuberculosis symptoms included cough, fever, and night sweats, while 72 (18.4%) did not. Lastly, 258 (65.8%) were aware that sputum smear microscopy is the most accurate method of tuberculosis diagnosis, while 134 (34.2%) were unaware as shown in Table 2.

**Table 2:** Demonstrates awareness of tuberculosis among participants. Awareness of tuberculosis (TB) among study participants, including general knowledge, understanding of symptoms, transmission methods, diagnosis, and prevention. Data is expressed as frequency (n) and percentage (%).

Parameters			n (%)
General knowledge	Have you heard about tuberculosis?	I am aware	371 (94.6)
		I am not aware	21 (5.4)
	Tuberculosis is an infectious disease that usually affects the lung.	I am aware	334 (85.2)
		I am not aware	58 (14.8)
	Mycobacterium tuberculosis bacteria is the cause of tuberculosis.	I am aware	270 (68.9)
		I am not aware	122 (31.1)
	Cough is a symptom of tuberculosis.	I am aware	347 (88.5)
		I am not aware	45 (11.5)
	Tuberculosis can spread from one person to another.	I am aware	335 (85.5)
		I am not aware	57 (14.5)
	There is a special test to diagnose tuberculosis?	I am aware	314 (80.1)
		I am not aware	78 (19.9)
	Tuberculosis is curable.	I am aware	304 (77.6)
		I am not aware	88 (22.4)
	Bacillus Calmette-Guérin (BCG) vaccine can prevent tuberculosis.	I am aware	296 (75.5)
		I am not aware	96 (24.5)
	Lungs are the most common organ affected by tuberculosis.	I am aware	339 (86.5)
		I am not aware	53 (13.5)
	Tuberculosis affects not only the lungs but also the lymph glands, bones, joints, brain, urinary tract, and reproductive system.	I am aware	275 (70.2)
		I am not aware	117 (29.8)
	Tuberculosis symptoms include cough, fever, night sweats, weight loss, shortness of breath, and chest pain.	I am aware	320 (81.6)
		I am not aware	72 (18.4)
	Sputum smear microscopy is the most accurate method of tuberculosis diagnosis.	I am aware	258 (65.8)
		I am not aware	134 (34.2)
Perception and attitude	Tuberculosis is a chronic disease.	I am aware	354 (90.3)
		I am not aware	38 (9.7)
	There is a treatment for tuberculosis?	I am aware	293 (74.7)
		I am not aware	99 (25.3)
	Acquired immunodeficiency syndrome is the greatest risk factor for developing active tuberculosis disease.	I am aware	251 (64)
		I am not aware	141 (36)
	Smoking can cause tuberculosis.	I am aware	236 (69.2)
		I am not aware	156 (39.8)
Barriers to TB care	Tuberculosis can be prevented	I am aware	294 (75)
		I am not aware	98 (25)
	Using a handkerchief or a towel while coughing will prevent the spread.	I am aware	304 (77.6)
		I am not aware	88 (22.4)
	The average life expectancy of an untreated HIV-positive individual who contracts tuberculosis is approximately six weeks.	I am aware	213 (54.3)
		I am not aware	179 (45.7)
	Tuberculosis can be spread through the air when an infected person coughs or sneezes.	I am aware	325 (82.9)
		I am not aware	67 (17.1)
	The duration of tuberculosis treatment is 6-9 months.	I am aware	241 (61.5)
		I am not aware	151 (38.5)

Regarding the association between gender and tuberculosis awareness, no significant difference was found, with males (128, 43%) having more awareness than females (170, 57%), while the p-value was 0.910 (not significant). When analyzing nationality, 192 (64.4%) Saudis had good awareness compared to 106 (35.6%) non-Saudis, with a p-value of 0.161 (not significant). For age, there was a borderline significant difference (p=0.058). Participants aged 18-22 had the highest level of poor awareness, with 40 (42.6%) compared to 88 (29.5%) having good awareness, whereas in the 40^+^ group, 76 (25.5%) had good awareness and 14 (14.9%) had poor awareness. Regarding marital status, there was a significant association with awareness (p=0.001). Single participants had the highest poor awareness rate, with 51 (54.3%) compared to 120 (40.3%) with good awareness. In contrast, married participants had 42 (44.7%) poor awareness and 126 (42.3%) good awareness. Divorced participants showed very low poor awareness (1, 1.1%) with 27 (9.1%) having good awareness, and no widowed participants demonstrated poor awareness. For occupation, there was a significant association (p=0.001). Health providers had the highest good awareness, with 85 (28.5%) participants, and only 11 (11.7%) showing poor awareness. Among housewives, 58 (19.5%) had good awareness and 22 (23.4%) had poor awareness. 46 (15.4%) engineers showed good awareness, and 11 (11.7%) had poor awareness. Among business professionals, 42 (14.1%) demonstrated good awareness and 9 (9.6%) had poor awareness. Lastly, among participants categorized as "other" 67 (22.5%) had good awareness, while a large portion (41, 43.6%) showed poor awareness. In summary, there was a significant association between tuberculosis awareness and marital status (p=0.001) as well as occupation (p=0.001), while age showed a borderline significance (p=0.058). Other variables, such as gender and nationality, did not show a significant association with awareness (Table 3).

**Table 3:** Association of awareness levels with demographic variables. Association of awareness levels with demographic variables among study participants, including gender, nationality, age, marital status, and occupation. Data is presented as frequency (n), percentage (%), and p-values. Statistically significant p-values (p<0.05) were observed for marital status (p=0.001) and occupation (p=0.001).

Parameters		Good (%)	Poor (%)	p-value	Chi-square value
Gender	Male	128 (43)	41 (43.6)	0.91	0.013
	Female	170 (57)	53 (56.4)		
	Total (%)	298 (76)	94 (24)		
Nationality	Saudi	192 (64.4)	53 (56.4)	0.161	1.97
	Non-Saudi	106 (35.6)	41 (43.6)		
	Total (%)	298 (76)	94 (24)		
Age	18-22	88 (29.5)	40 (42.6)	0.058	7.47
	23-29	72 (24.2)	20 (21.3)		
	30-39	62 (20.8)	20 (21.3)		
	40+	76 (25.5)	14 (14.9)		
	Total (%)	298 (76)	94 (24)		
Marital status	Single	120 (40.3)	51 (54.3)	0.001	17.58
	Married	126 (42.3)	42 (44.7)		
	Divorced	27 (9.1)	1 (1.1)		
	Widow	25 (8.4)	0 (0)		
	Total (%)	298 (76)	94 (24)		
Occupation	Housewife	58 (19.5)	22 (23.4)	0.001	22.2
	Engineer	46 (15.4)	11 (11.7)		
	Health provider	85 (28.5)	11 (11.7)		
	Business	42 (14.1)	9 (9.6)		
	Other	67 (22.5)	41 (43.6)		
	Total (%)	298 (76)	94 (24)		

## Discussion

This cross-sectional study assessed tuberculosis (TB) awareness among the general population in Riyadh, Saudi Arabia, revealing high general awareness levels, with 94.6% of participants having heard about tuberculosis, a finding consistent with studies conducted in other regions of Saudi Arabia and Oman [2,5]. However, significant gaps remain in specific knowledge areas. Only 80.1% of participants were aware of a diagnostic test for tuberculosis, and 31.1% did not recognize *Mycobacterium tuberculosis* as the causative agent, reflecting knowledge gaps similar to those reported in studies from Southeast China and Pakistan, where public health education initiatives have yet to close fundamental awareness gaps [6,7].

Furthermore, while 75.5% of participants acknowledged the preventive role of the Bacillus Calmette-Guérin (BCG) vaccine, 24.5% were unaware of its importance, highlighting a persistent global challenge in promoting vaccine awareness. Similar findings have been reported in studies conducted in the Middle East, Southeast Asia, and Nepal, where misconceptions about tuberculosis prevention remain widespread [5,10,13]. Misunderstandings about diagnostic methods were also evident, with 34.2% of participants unaware of the accuracy of sputum smear microscopy, emphasizing the need for stronger diagnostic literacy. Such gaps align with findings from studies conducted in Lesotho and urban slums in India, where knowledge about diagnostic tools was found to be limited [8,13]. This study also revealed significant associations between awareness levels and demographic factors. Married individuals showed greater awareness compared to single participants, which may reflect better engagement with healthcare systems, a pattern observed in studies from Pakistan and Jeddah [4,7].

Healthcare workers demonstrated the highest awareness levels (28.5%), which is consistent with findings globally, underscoring the critical role of professional exposure to health education in boosting awareness [3,8]. In contrast, housewives and individuals in non-healthcare occupations exhibited lower awareness levels, reflecting disparities in access to health education and outreach programs, as reported in studies conducted in Ethiopia and Jeddah [4,11]. Younger participants, particularly those below 30 years of age, also demonstrated comparatively lower levels of tuberculosis knowledge, a trend noted in studies from Southeast Asia and Sub-Saharan Africa, where younger populations often have less exposure to structured health information [6,11]. When examining comparisons with international studies, awareness levels in Riyadh appear higher than those observed in Southeast China, where gaps in knowledge about transmission and prevention were highlighted [6].

However, they align closely with findings from Oman, emphasizing the importance of targeted educational programs [5]. In Pakistan and Ethiopia, lower awareness among rural populations has been attributed to limited access to healthcare services and information, a concern that may similarly affect rural areas in Saudi Arabia [7,11]. Social stigma surrounding TB remains a challenge, as noted in studies from Nepal and India, where fear of discrimination reduces willingness to seek care [10,14]. While this study did not directly measure stigma, the findings underscore the need for campaigns addressing misconceptions and reducing social barriers to TB care [10]. Awareness campaigns conducted during large gatherings, such as those in India, demonstrate the potential for improving public understanding in a short period, a strategy that could be adapted locally [14]. The findings of this study highlight the urgent need for targeted and culturally relevant health education campaigns, particularly aimed at underserved groups such as housewives and younger individuals. However, certain limitations must be acknowledged. This study relied on self-reported data, which is subject to potential recall and reporting biases, and its cross-sectional design precludes causal interpretations of the relationships between demographic factors and tuberculosis awareness. Furthermore, the study was limited to Riyadh, and findings may not fully reflect awareness levels in rural or underserved regions of Saudi Arabia, where access to health education and services is likely more restricted. Addressing these limitations in future studies, including longitudinal designs and expanded geographic coverage, could provide a more comprehensive understanding of tuberculosis awareness and its determinants across Saudi Arabia.

## Conclusions

This study provides valuable insights into the level of awareness and knowledge of tuberculosis among the general population in Riyadh, Saudi Arabia. While the overall awareness of tuberculosis is relatively high, there are critical gaps in knowledge regarding its transmission, diagnosis, and prevention. Misconceptions about tuberculosis remain, particularly in understanding its causes, diagnostic methods, and preventive measures like the Bacillus Calmette-Guérin vaccine. The significant associations between tuberculosis awareness and demographic factors, such as marital status and occupation, highlight the need for tailored public health interventions. Healthcare providers demonstrated higher awareness levels, indicating that professional exposure enhances tuberculosis knowledge. In contrast, housewives and individuals in non-healthcare occupations exhibited lower awareness, pointing to the necessity of targeted educational campaigns for these groups. The borderline significance found in younger age groups suggests that this demographic may also require focused health education efforts. In conclusion, public health strategies aimed at increasing tuberculosis knowledge should address the specific knowledge gaps identified in this study, ensuring that accurate information reaches all segments of the population. By improving awareness, particularly around prevention and diagnosis, these efforts can contribute to better tuberculosis control and prevention in Saudi Arabia.

## Appendices

Appendix A

**Table 4:** Tuberculosis awareness questionnaire.

#	Question	Response
1	Have you heard about tuberculosis?	☐ I'm aware ☐ I'm not aware
2	Is tuberculosis a fatal disease?	☐ I'm aware ☐ I'm not aware
3	TB is an infectious disease that usually affects the lungs.	☐ I'm aware ☐ I'm not aware
4	*Mycobacterium tuberculosis* bacteria is the cause of TB.	☐ I'm aware ☐ I'm not aware
5	Cough is a symptom of tuberculosis.	☐ I'm aware ☐ I'm not aware
6	Tuberculosis can spread from one person to another.	☐ I'm aware ☐ I'm not aware
7	Is there a special test to diagnose tuberculosis?	☐ I'm aware ☐ I'm not aware
8	Tuberculosis is curable.	☐ I'm aware ☐ I'm not aware
9	Is there a treatment for TB?	☐ I'm aware ☐ I'm not aware
10	Can TB be prevented?	☐ I'm aware ☐ I'm not aware
11	Using a handkerchief or a towel while coughing will prevent the spread.	☐ I'm aware ☐ I'm not aware
12	The BCG vaccine can prevent TB.	☐ I'm aware ☐ I'm not aware
13	The average lifespan of an untreated HIV-infected person who contracts TB is 6 weeks.	☐ I'm aware ☐ I'm not aware
14	AIDS is the greatest risk factor for developing active TB disease.	☐ I'm aware ☐ I'm not aware
15	TB can be spread through the air when an infected person coughs or sneezes.	☐ I'm aware ☐ I'm not aware
16	The lungs are the most common organ affected by TB.	☐ I'm aware ☐ I'm not aware
17	TB affects only the lungs, lymph glands, bones, joints, brain, urinary tract, and reproductive system.	☐ I'm aware ☐ I'm not aware
18	TB symptoms include cough, fever, night sweats, weight loss, shortness of breath, and chest pain.	☐ I'm aware ☐ I'm not aware
19	The duration of TB treatment is 6-9 months.	☐ I'm aware ☐ I'm not aware
20	Does smoking cause TB?	☐ I'm aware ☐ I'm not aware
21	Sputum smear microscopy is the most accurate method of TB diagnosis.	☐ I'm aware ☐ I'm not aware
